# Association between Psychological Resilience and Self-Rated Health in Patients with Knee Osteoarthritis

**DOI:** 10.3390/healthcare11040529

**Published:** 2023-02-10

**Authors:** Chun-Man Hsieh, Aih-Fung Chiu, Chin-Hua Huang

**Affiliations:** 1 Department of Nursing, Tajen University, Pingtung 90741, Taiwan; 2 Department of Nursing, Meiho University, Pingtung 91202, Taiwan; 3 Department of Information Engineering, I-Shou University, Kaohsiung 84001, Taiwan; 4 Department of Health Business Administration, Meiho University, Pingtung 91202, Taiwan

**Keywords:** 10-item Connor–Davidson Resilience Scale (CD–RISC-10), resilience, knee osteoarthritis, self-rated health, Western Ontario and McMaster Universities Osteoarthritis Index (WOMAC)

## Abstract

This study aimed to evaluate whether psychological resilience is an independent factor of self-rated health (SRH) among patients with knee osteoarthritis (KOA). A cross-sectional study with convenience sampling was designed. Patients with doctor-diagnosed KOA were recruited from the orthopedic outpatient departments of a hospital in southern Taiwan. Psychological resilience was measured by the 10-item Connor–Davidson Resilience Scale (CD–RISC-10), and SRH was measured by three items, including the current SRH, the preceding year-related SRH, and age-related SRH. The three-item SRH scale was categorized as “high” and “low–moderate” groups by terciles. Covariates included KOA history, site of knee pain, joint-specific symptoms measured by the Western Ontario and McMaster Universities Osteoarthritis Index (WOMAC), comorbidity measured by Charlson Comorbidity Index, and demographic variables (i.e., age, sex, education attainment, and living arrangements). A multiple logistic regression was used to detect the independent variables with significant odds ratios that can predict “high” SRH among participants. Results: In total, 98 patients with KOA (66 women and 32 men) with a mean age (±SD) of 68.3 ± 8.5 years were enrolled and were analyzed. A total of 38.8% (n = 38) of participants were categorized as “high SRH”, while 61.2% (n = 60) were categorized as “low–moderate SRH”. Multiple logistic regression showed that CD–RISC-10 had an increased odds ratio (OR) for high SRH (OR [95% CI] = 1.061 [1.003–1.122]; *p* = 0.038), whereas bilateral pain (vs. unilateral pain), WOMAC stiffness, and WOMAC physical limitation showed a decreased OR for high SRH (0.268 [0.098–0.732], 0.670 [0.450–0.998], and 0.943 [0.891–0.997], respectively). Our findings provide evidence indicating that psychological resilience plays a significant positive role in the SRH in our study sample. Further research is required to extend the growing knowledge regarding the application of psychological resilience on KOA.

## 1. Introduction

An aging society and the prevalence of chronic diseases highlight people’s need for good health. In order to improve the health of people, healthcare providers are always committed to seeking corresponding solutions. In addition to medications and lifestyle changes, healthcare providers are increasingly aware of the potential contribution of positive psychological factors to chronic disease management. Psychological resilience is an important topic that is discussed in the literature. The concept of resilience is originally derived from technical sciences and refers to the ability of a material to recover or rebound to its original shape after bending or compression. Psychological resilience has gradually become prevalent in the fields of psychology and psychiatry and has recently been garnering attention in the field of chronic illness.

Psychological resilience has two distinct meanings. On the one hand, resilience can be narrowly described as a trait or a personality resource, an individual’s ability to resist being damaged or deformed by traumas or destructive forces. It enables individuals to adjust and modify habitual expression patterns of self-discipline and self-control so that they can face and adapt to the situation in the present and future. On the other hand, resilience broadly means readily “bouncing back” or recovering from those traumas or destructive forces. That is, when an individual is facing adversity, they can go further than merely coping by finding meaning in the trauma-inducing events, and they can further utilize this meaning to enhance their well-being to achieve the status of post-traumatic growth [1,2]. 

As an integrated concept of resilience, the American Psychological Association Dictionary of Psychology’s advanced definition defines resilience as “the process and outcome of successfully adapting to difficult or challenging life experiences, especially through mental, emotional, and behavioral flexibility and adjustment to external and internal demands” [3]. This means that through mental processes and behaviors, resilience can promote or protect individuals from the potentially negative effects of stress or adversity. In terms of resources or constructs, psychological resilience can be treated as a single concept [4] or as a composite of several distinct traits or capacities [1,5]. A wide variety of candidate constructs, as either antecedents or components, have been suggested for inclusion in the concept of resilience, including acceptance of change, control, personal competence, spiritual influences, persistence/tenacity, self-efficacy, emotion regulation, optimism, adaptability/ability to bounce back, and social support [1,5]. 

In recent decades, increasing attention is being paid to resilience in response to chronic illness. In this context, psychological resilience has been described as the flexibility and ability to cope, recover, or adapt when facing internal and external pressures brought on by a chronic illness [6,7]. As a protective factor, psychological resilience has been confirmed to have great potential in facilitating the health outcome in people with chronic illnesses [8,9], such as different types of cancer [10,11], cardiovascular disease [12,13], and even chronic low back pain [13]. However, no comparable studies have been carried out with knee osteoarthritis (KOA).

KOA is a prevalent chronic illness and an age-related degenerative joint disease. KOA cannot be completely healed using medicine and usually requires a long period of supervision, observation, and management [14]. KOA symptoms, such as joint pain, aching, stiffness, or limited function, often negatively impact overall health [15]. People with osteoarthritis tend to rate their health as poor [16]. Based on the health promotion of patients with KOA, it is necessary to understand the relevant attributes of health. Resilience is a positive psychological factor that can potentially promote health. However, previous works examining the correlation of health outcomes for KOA patients are usually focused on other variables, rather than psychologic resilience. It remains unclear whether subjects with a high level of psychological resilience would experience better health. 

Self-rated health (SRH) is the result of a cognitive assessment of the integrated biological, mental, social, and functional aspects of an individual [17,18]. The World Health Organization recommends SRH as a good indicator of overall health status that can be used as a proxy for a multi-dimensional concept of health [19]. Evidence-based studies have confirmed that SRH is significantly associated with multiple domains of health and can be used as a useful summary indicator for monitoring health status at a population level [17,20,21]. Additionally, SRH can reliably predict mortality among middle-aged or older people [22,23,24]. In terms of correlations, SHR was reported to be significantly associated with clinical parameters, such as medical conditions, laboratory findings, or functional activity or limitations [16,25,26,27], and psychological factors such as depression [25,26]. Demographic characteristics such as age, sex, marital status, or education attainment may also be factors that are directly or indirectly associated with SRH [25,26,27,28]. For the purposes of health promotion, directly focusing on favorable health outcomes such as SRH is needed. Healthcare providers who monitor SRH in their patients can understand the effects of health promotion interventions, provide timely assistances, and contribute to the maintenance of health. In addition, nursing care is critical to developing and applying effective strategies to promote psychological resilience for patients with KOA. Based on the above discussion, the present study is focused on SRH as an outcome and examined the association of psychological resilience and other related variables with a high SRH among people with KOA. 

### Study Hypothesis

With a special interest in this positive psychological trait, we hypothesized that patients exhibiting greater psychological resilience would report a better SRH. 

## 2. Methods

### 2.1. Study Design and Sample

A cross-sectional study with convenience sampling was adopted, and participants were recruited from the orthopedic outpatient departments of a hospital in southern Taiwan from July to August 2020. Subjects were eligible for the study if they met the following criteria: (1) patients with doctor-diagnosed KOA, (2) those who agreed to participate in the study and provided informed consent, and (3) those with clear consciousness, no cognitive impairment, or other severe diseases that would limit their ability to complete a survey. Patients with a knee joint replacement or who were diagnosed with rheumatologic diseases were excluded. A total of 98 KOA patients participated in this study.

According to the recommendations of Concato et al. and Peduzzi et al., the minimum required sample size should be based on the rule of event per variable (EPV) and the concept of an EPV of 10 is acceptable for logistic regression [29,30]. Therefore, the total 98 participants in the present study sample size met the requirement. 

### 2.2. Procedure

Researchers and two trained research assistants met the participants before or after their physician appointments at the orthopedic outpatient departments of a hospital in southern Taiwan. After the interviewers informed them of the study’s purpose and obtained written informed consent, face-to-face interviews were conducted. All participants were able to withdraw from the study and withdraw their consent at any time. Ethical approval was obtained from the institutional review committee of the hospital. 

### 2.3. Measures

The questionnaire included psychological resilience measured by the 10-item Connor–Davidson Resilience Scale (CD–RISC-10), SRH, and covariates (sociodemographic and clinical characteristics). 

#### 2.3.1. Psychological Resilience

The Chinese version of the CD–RISC-10 is widely used to assess psychological resilience [31,32], specifically the ability to cope with adversity [5]. This scale contains diverse items corresponding to flexibility (2 items), sense of self-efficacy (3 items), ability to regulate emotion (1 item), optimism (3 items), and cognitive focus/maintaining attention under stress (1 item) [31]. Each item is measured on a 5-point scale ranging from 0 (not true at all) to 4 (true nearly all the time). The potential total score ranges from 0 to 40, with higher scores indicating greater resilience. The available evidence supports its construct validity when administered to older adults or to medical patients [31,33,34]. In the present study, the CD–RISC-10 scale represented a high reliability of internal consistency with a Cronbach’s alpha of 0.945.

#### 2.3.2. Self-Rated Health (SRH)

SRH is a measure of how people perceive their health [35]. In the literature, three different operational definitions and three items were found [36,37]. These are the current SRH, SRH compared to 1 year ago (the preceding year-related SRH) [36], and SRH compared to other people of their age (age-related SRH) [37]. Specifically, item 1: “In general, how would you rate your current health condition?”, with a range of response options of “excellent”, “very good”, “good”, “fair”, and “poor”; item 2: “How would you rate your health compared to 1 year ago?” with response options of “much better”, “somewhat better”, “about the same”, “somewhat worse”, or “much worse”; and item 3: How would you rate your health when comparing with someone of your age”, with response options of “much better”, “somewhat better”, “about the same”, “somewhat worse”, or “much worse”. The former 2 items were the same ones from the general health perception subscale of the Chinese version of the 36-item Short Form Health Survey [36]. Unlike the others, item 3 was commonly used to represent individual health when comparing people in the same age range [37]. Each of the three items was rated on a 5-point Likert scale (1 to 5). The total potential score ranged from 3 to 15 points, with a higher SRH score indicating a better health status.

In the present study, the content validity index of clarity (panel reviewed by experts) for this 3-item SRH scale was 1.00. The Cronbach’s alpha was 0.620. The reliability of the test is relatively low; however, it can be accepted [38]. The SRH, as a dependent variable, did not have a normal distribution and was skewed toward the higher range. Consequently, participants were divided into two groups. Participants within the upper tercile of the total SRH scores (SRH score ≥ 9) were arbitrarily dichotomized into a “high” SRH group, while the others were dichotomized into a “low–moderate” SRH group (SRH score < 9). 

#### 2.3.3. Covariates

Based on previous studies on SRH, the following covariates were considered in this study. The sociodemographic characteristics included age, gender, education attainment (elementary school and under, junior or senior high school, and college or above), living arrangements (with/without a spouse), self-reported KOA history (<5 years, ≥5 years), site of knee pain (unilateral or bilateral), KOA symptoms, depression, and comorbidity. 

KOA symptoms were assessed using the Western Ontario and McMaster Universities Osteoarthritis (WOMAC) Index, which was developed in 1982 at the Western Ontario and McMaster Universities. The WOMAC Index includes 3 dimensions: stiffness (2 items), pain (5 items), and physical function (17 items). All items are scored on a scale of 0 to 4, corresponding to “none”, “mild”, “moderate”, “severe”, and “extreme”, respectively. Higher scores indicate greater severity of symptoms [39]. WOMAC is a valid tool for evaluating joint-related symptoms and has been linguistically validated [40]. In the present study, Cronbach’s alpha reliability for total and its three dimensions (stiffness, pain, and physical functioning) were 0.948, 0.796, 0.795, and 0.994, respectively. Comorbidity was self-reported using the Charlson Comorbidity Index (CCI) [41]. Depression status was assessed using the 5-item Geriatric Depression Scale (GDS-5), where one point is assigned to each “yes” response and a total score ≥ 2 indicates the presence of depression [42]. In the present study, the internal consistency of the GDS-5 scale was acceptable, with a Cronbach’s alpha of 0.673 [38]. 

### 2.4. Data Analysis

Descriptive statistics are reported as a percentage for categorical variables and as a mean ± standard deviation (SD) for continuous variables. Because most of the data did not follow a normal distribution, nonparametric analyses (Chi-square tests and Mann–Whitney U tests) were used to compare the demographic and clinical characteristics, as well as psychological resilience between the two levels of SRH (low–moderate and high SRH). If there were significant variables that were found between the two levels of SRH, the univariate logistical regression was conducted to assess the relationship between psychological resilience and SRH without considering covariates (univariate model). Multiple logistical regression with the conditional method was then used, controlling for all covariates (adjusted model). The results are reported as odds ratios (ORs) or adjusted odds ratio (AOR) with 95% confidence intervals (CIs). In terms of internal consistency, Cronbach’s alpha coefficients were calculated for all the scales. According to Guilford’s (1965) criteria, if Cronbach’s α value is greater than 0.7, then it is considered a good reliability value, while values between 0.7 and 0.35 are considered acceptable reliability values. Values less than 0.35 are rejected reliability values [38]. The main analysis tests the association of psychologic resilience with SRH using multiple logistic regression with the conditional method. Thus, the odds ratio of the logistic regression model can be used as an effect size statistic. Data analyses were conducted using the IBM SPSS statistical package version 25.0 (SPSS; Chicago, IL, USA). All statistical analyses were two-tailed and a *p*-value less than 0.05 was considered statistically significant.

## 3. Results

### 3.1. Participant Characteristics

A total of 98 people with KOA, consisting of 32 men and 66 women, were enrolled in this study. The mean age (SD) was 68.3 ± 8.5 years, with a range of 51–90 years. In SRH, the median SRH score was 8 (range: 3–14). Using the upper tercile of the total SRH score (score = 9) as the cut-off point, 38 (38.8%) patients were dichotomized into “high SRH” (score ≥ 9) group, while 60 (61.2%) participants were dichotomized into “low–moderate” SRH (score < 9) group. 

### 3.2. Binary Logistic Regression Analysis

When compared with the high SRH group, participants in the low–moderate SRH group reported a significantly higher proportion (*p* < 0.05 for all comparisons) of a long history of KOA (≥5 years), bilateral knee pain (vs. unilateral), and higher comorbidities (CCI < 1). In addition, participants in the low–moderate SRH group had higher scores in the total WOMAC index and its subscales (severe KOA symptoms), and lower scores in CD–RISC-10 (*p* < 0.05). However, there were no significant differences found in the proportion of the other variables between both SRH groups (Table 1). Table 1 outlines the participants’ characteristics between the two levels of SRH among the study sample (n = 98).

### 3.3. Association of WOMAC Score and CD–RISC-10 Score with High Self-Rated Health

Table 2 showed the results of the univariate logistic model. Self-reported OA history (<5 years or ≥5 years), site of pain knee (uni- or bilateral knee pain), comorbidity (CCI ≥ 1 or <1), the total and subscales of WOMAC index were negatively related to high SRH, while CD–RISC-10 score were positively related to high SRH (all *p* < 0.05). 

Table 3 shows the result of a multiple logistic regression with the conditional method. After adjusting for all covariates (self-reported OA history, site of pain knee, comorbidity, WOMAC index score, and CD–RISC-10 score), participants with bilateral pain had a decreased AOR (OR = 0.268, 95% CI = 0.098–0.732) with a high SRH, compared to those with unilateral pain. In addition, each 1-point increase in scores of WOMAC stiffness and WOMAC physical limitation decreased the AOR of reporting a high SRH (OR = 0.670, 95% CI = 0.450–0.998, and AOR = 0.943, 95% CI = 0.891–0.997, respectively). On the other hand, each 1-point increase in the CD–RISC-10 score increased the AOR of reporting a high SRH (OR = 1.061, 95% CI = 1.003–1.122, and *p* = 0.038). This result implies that KOA-related symptoms are negatively associated with a high SRH, and that psychological resilience was a positive predictor of SRH. 

All tests on the Wald values for bilateral pain, WOMAC stiffness, WOMAC physical limitation, and the CD–RISC-10 score, resulted in *p*-values < 0.05, indicating that these four variables are significant predictors of SRH in people with KOA. The regression model had a high goodness-of-fit, with Omnibus test of model coefficients χ^2^ = 26.304, df = 4 (*p* < 0.001), indicating that this predictive model was able to distinguish between participants who did and did not report a high SRH. In terms of the strength of association, the model explained approximately 23.5–31.9% of the variance in SRH (range between Cox and Snell R^2^ and Nagelkerke R^2^).

## 4. Discussion

The present study showed that psychological resilience was a key determinant of a high SRH among patients with KOA. After controlling the other significant variables, the higher the CD–RISC-10 scores, the higher the chance of reporting a high SRH in our study sample (Table 3). Conversely, higher symptoms tended to be associated with lower odds of reporting a high SRH. 

To our knowledge, this is the first study to determine the association of psychological resilience with a high SRH among patients with KOA. This finding is congruent with the results reported in similar studies. A cross-sectional study surveyed 163 patients following a primary total knee arthroplasty and reported that psychological resilience measured by CD–RISC-10 was identified as a significant predictor of functional outcome [43]. In addition, there were two studies that used a different resilience scale. In one study, they followed 153 patients with a total knee arthroplasty and found that pre-operative resilience can predict overall physical and mental health outcomes, 3 and 12 months later [44]. Another study targeting 117 patients with a total knee arthroplasty reported that pre-operational resilience was positively associated with a better post-operative knee function and better general physical health 3 months later, but it was not the case for general mental health [45]. In the present study, with the measurement of psychological resilience using the CD–RISC-10 and perceived health status using a 3-item SRH scale, our research further confirms the importance of psychological resilience on health status among patients with KOA. 

In the literature, psychological resilience has been considered as a kind of cognitive–affective state and can modify or alleviate perceptual chronic pain through the activation and inflammation of the immune system [9]. Low psychological resilience, as driving mechanisms, can accelerate the development of chronic aging diseases, through increased inflammation, oxidative stress, and chronic medical conditions [46]. In addition, KOA is a degenerative and inflammatory chronic disease. Hence, we supposed that to a certain extent, the biochemical mechanisms of inflammation may exist in part to explain the relationship between psychological resilience and high health status among patients with KOA. However, further studies are required to explore and examine the mechanisms linking psychological resilience with health status. 

Our significant finding represents opportunities for healthcare providers to give resilience-based interventions or strategies for patients with KOA. Specifically, healthcare providers can enable KOA patients to enhance their psychological resilience through the development or cultivation of resilience constructs or protective factors, such as a sense of self-efficacy, the ability to regulate emotions, coping mechanisms, or through creating a supportive environment [47]. Once patients have the sufficient resilience, they would have the ability to adapt or cope with the suffering from KOA to achieve better health and well-being.

In the present study, we measured health status using a 3-item SRH scale, which was derived from the previous study, and investigated the association with psychological resilience. Given that the SRH is a simple and generally feasible method with acceptable validity, we used it as a measurement for the assessment of health status. The upper tercile of the total SRH score was used as a cut-off threshold (score = 9) for high SRH. Though high SRH does not guarantee health, it is indeed significantly associated with objective health measures and consequent morbidity [18]. Due to its simplicity and feasibility, the SRH scale may be a suitable proxy for surveying the health of the community, even in clinical settings. In addition, according to the principles of patient-centered care, the patient’s perspective is important; people with low SRH tend to suffer from more illnesses or symptoms and need further medical attention [48]. 

As expected, the KOA-related symptoms (i.e., bilateral vs. unilateral knee pain, WOMAC stiffness, and WOMAC physical limitation) showed a negative relationship with high SRH. Physical health or illness-related factors, such as existing medical conditions, pain severity, functional problems, or disabilities, have also been reported to be strong predictors of poor SRH [26,27]. Given that SRH is based on an individual’s subjective perception of their physical health, people with severe KOA-related symptoms are more likely to rate their health as poor. Methods of prevention or treatment interventions for KOA should be further developed to improve SRH among people with KOA symptoms. 

The present study sheds light on the relationship of some variables with SRH, especially the positive effects of psychological resilience, but it is still subject to some limitations. Firstly, due to the cross-sectional design of our study, the causality between relationships should be carefully interpreted. Advanced research to employ experimental and longitudinal designs may be required to determine if and how psychological resilience impacts SRH and to examine the interaction between KOA symptoms and SRH. Secondly, the relatively small sample size of this study (n = 98) not only limits the generalization scope but also makes Cronbach’s alpha of SRH and GDS-5 relatively low (SRH for 0.620 and GDS-5 for 0.673, respectively) [49]. Accordingly, it may be important to perform future studies to replicate the current findings with a larger sample size. Finally, only 23.5–31.9% of the variance in SRH scores was explained by the multiple logistic regression model, implying that there are other variables that remain unelucidated and may explain the remaining variance. Despite these limitations, our study provides useful information for policy-makers, health planners, and healthcare providers by addressing the importance of resilience on SRH among people with KOA. 

## 5. Conclusions

Our findings contribute to the growing body of research regarding the specific role of psychological resilience in high SRH among patients with KOA. Healthcare providers can devise and tailor preventive interventions that target psychological resilience to promote better health among patients with KOA. Future studies utilizing experimental designs may also be needed to elucidate the effectiveness of resilience on a person’s health.

## Figures and Tables

**Table 1:** Participants’ characteristics between two levels of SRH among study sample (n = 98).

	Low–Moderate SRH (Score < 9, n = 60)	High SRH (Score ≥ 9, n = 38)		
Variables	N (%)	N (%)	χ^2^ (f)/t (f)	*p*-Value
Gender				
Men (n = 32)	18 (30.0)	14 (36.8)	0.95 (1)	0.482
Women (n = 66)	42 (70.0)	24 (63.2)		
Age (Mean ± SD)	68.0 ± 8.8	68.8 ± 8.0	−0.42 (96)	0.673
Education attainment				
Elementary school and under (n = 33)	22 (36.7)	11 (28.9)	1.88 (2)	0.390
Junior or senior high school (n = 51)	28 (46.7)	23 (60.5)		
College or above (n = 14)	10 (16.7)	4 (10.5)		
Living conditions				
With spouse (n = 40)	27 (45.0)	13 (34.2)	1.12 (1)	0.290
Without spouse (n = 58)	33 (55.0)	25 (65.8)		
Self-reported OA history				
<5 years (n = 51)	25 (41.7)	26 (68.4)	6.67 (1)	0.010 *
≥5 years (n = 7)	35 (58.3)	12 (31.6)		
Site of pain knee				
Unilateral pain (n = 55)	27 (45.0)	28 (73.7)	7.77 (1)	0.005 *
Bilateral pain (n = 43)	33 (55.0)	10 (26.3)		
Comorbidity				
CCI < 1 (n = 62)	33 (55.0)	29 (76.3)	4.55 (1)	0.033 *
CCI ≥ 1 (n = 36)	27 (45.0)	9 (23.7)		
GDS-5				
<2 (n = 83)	48 (80.0)	48 (80.0)	2.63 (1)	0.105
≥2 (n = 15)	12 (20.0)	12 (20.0)		
	**Low–Moderate SRH** **(Score < 9, n = 60)**	**High SRH** **(Score ≥ 9, n = 38)**		
**Variables**	**Mean ± SD**	**Mean ± SD**	**Mann–Whitney U**	***p*-Value**
Total WOMAC score	46.5 ± 14.8	36.6 ± 10.5	552.00	<0.001 *
WOMAC stiffness	4.0 ± 1.6	3.2 ± 1.0	820.50	0.019 *
WOMAC pain	10.0 ± 3.4	8.3 ± 2.8	608.50	0.006 *
WOMAC physical limitation	32.5 ± 11.3	25.1 ± 8.4	546.50	<0.001 *
CD–RISC-10 score	25.2 ± 9.6	29.4 ± 8.5	673.00	0.036 *

CCI, Charlson Comorbidity Index; CD–RISC-10, 10-items Connor–Davidson Resilience Scale; GDS, Geriatric Depression Scale; SRH, self-rated health; and WOMAC Index, McMaster Universities Osteoarthritis Index. χ^2^ -tests and Mann–Whitney U test were used for categorical variables and for continuous variables, respectively. * *p* < 0.05

**Table 2:** Univariate logistic regression for high self-rated health in people with KOA (n = 98).

Variables	OR	(95% CI)	Wald Value (df)	*p*-Value
Self-reported OA history				
≥5 years (reference: <5 years)	0.330	(0.140–0.775)	6.468 (1)	0.011 *
Site of pain knee (reference: unilateral pain)				
Bilateral pain	0.292	(0.121–0.707)	7.454 (1)	0.006 *
Comorbidity				
CCI ≥ 1(reference: CCI < 1)	0.379	(0.154–0.937)	4.413 (1)	0.036 *
Total WOMAC score	0.937	(0.900–0.975)	10.365 (1)	0.001 *
WOMAC stiffness		(0.466–0.902)	6.613 (1)	0.010 *
WOMAC pain	0.822	(0.703–0.960)	6.116 (1)	0.013 *
WOMAC physical limitation	0.921	(0.874–0.970)	9.622 (1)	0.002 *
CD–RISC-10	1.052	(1.004–1.104)	4.431 (1)	0.035 *

* *p* < 0.05. CI, confidence interval; df, degrees of freedom; and OR, odds ratio.

**Table 3:** Multiple logistic regression analysis with conditional method for high self-rated health in people with KOA (n = 98).

Variables	AOR	(95% CI)	Wald Value (df)	*p*-Value
Site of pain knee (reference: unilateral pain)				
Bilateral pain	0.268	(0.098–0.732)	6.583 (1)	0.010 *
Total WOMAC score				
WOMAC stiffness	0.670	(0.450–0.998)	3.881	0.049 *
WOMAC physical limitation	0.943	(0.891–0.997)	4.200	0.040 *
CD–RISC-10	1.061	(1.003–1.122)	4.286	0.038 *
Constant	1.204		1.204	0.882

* *p* < 0.05. CI, confidence interval; df, degrees of freedom; and AOR, adjust odds ratio for all variables included in the regression model. Omnibus test of model coefficients χ^2^ = 26.304, df = 4, *p* < 0.001. Strength of association: Cox and Snell R^2^ = 0.235; Nagelkerke R^2^ = 0.319.

## Data Availability

Data is unavailable due to privacy or ethical restrictions.

## References

[1] Harms P.D., Brady L., Wood D., Silard A., Diener E., Oishi S., Tay L. (2018). Resilience and well-being. Handbook of Well-Being.

[2] Fletcher D., Sarkar M. (2013). Psychological resilience: A review and critique of definitions, concepts, and theory. Eur. Psychol..

[3] American Psychological Association Resilience. https://dictionary.apa.org/resilience.

[4] Smith B.W., Dalen J., Wiggins K., Tooley E., Christopher P., Bernard J. (2008). The brief resilience scale: Assessing the ability to bounce back. Int. J. Behav. Med..

[5] Connor K.M., Davidson J.R.T. (2003). Development of a new resilience scale: The Connor-Davidson Resilience Scale (CD-RISC). Depress. Anxiety.

[6] Babić R., Babić M., Rastović P., Ćurlin M., Šimić J., Mandić K., Pavlović K. (2020). Resilience in health and illness. Psychiatr. Danub..

[7] Kim G.M., Lim J.Y., Kim E.J., Park S. (2018). Resilience of patients with chronic diseases: A systematic review. Health Soc. Care Community.

[8] Bartley E.J., Palit S., Staud R. (2017). Predictors of Osteoarthritis Pain: The Importance of Resilience. Curr. Rheumatol. Rep..

[9] Sturgeon J.A., Zautra A.J. (2010). Resilience: A New Paradigm for Adaptation to Chronic Pain. Curr. Pain Headache Rep..

[10] Macía P., Barranco M., Gorbeña S., Álvarez-Fuentes E., Iraurgi I. (2021). Resilience and coping strategies in relation to mental health outcomes in people with cancer. PLoS ONE.

[11] Seiler A., Jenewein J. (2019). Resilience in Cancer Patients. Front. Psychiatry.

[12] M Al Ali N., Al Ramamneh I.S. (2021). Resilience of patients with coronary heart diseases in Jordan: A cross-sectional study. Int. J. Nurs. Sci..

[13] Wang X., Gao J., Zhang J., Yang Y., Zhang W., Zhang X., Lu L., Wang R. (2022). Factors associated with psychological resilience in patients with chronic heart failure and efficacy of psycho-cardiology intervention. Am. J. Transl. Res..

[14] Kolasinski S.L., Neogi T., Hochberg M.C., Oatis C., Guyatt G., Block J., Callahan L., Copenhaver C., Dodge C., Felson D. (2020). 2019 American College of Rheumatology/Arthritis Foundation Guideline for the Management of Osteoarthritis of the Hand, Hip, and Knee. Arthritis Rheumatol..

[15] Vitaloni M., Bemden A.B.-V., Contreras R.M.S., Scotton D., Bibas M., Quintero M., Monfort J., Carné X., de Abajo F., Oswald E. (2019). Global management of patients with knee osteoarthritis begins with quality of life assessment: A systematic review. BMC Musculoskelet. Disord..

[16] Wu S., Wang R., Zhao Y., Ma X., Wu M., Yan X., He J. (2013). The relationship between self-rated health and objective health status: A population-based study. BMC Public Health.

[17] Garbarski D. (2016). Research in and Prospects for the Measurement of Health Using Self-Rated Health. Public Opin. Q..

[18] Jylhä M. (2009). What is self-rated health and why does it predict mortality? Towards a unified conceptual model. Soc. Sci. Med..

[19] De Bruin A., Picavet H.S., Nossikov A. (1996). Health interview surveys. Towards international harmonization of methods and instruments. WHO Reg. Publ. Eur. Ser..

[20] Jerković O.S., Sauliūnė S., Šumskas L., Birt C., Kersnik J. (2015). Determinants of self-rated health in elderly populations in urban areas in Slovenia, Lithuania and UK: Findings of the EURO-URHIS 2 survey. Eur. J. Public Health.

[21] Bunda K., Busseri M.A. (2019). Subjective Trajectories for Self-Rated Health as a Predictor of Change in Physical Health Over Time: Results from an 18-Year Longitudinal Study. Soc. Cogn..

[22] Wuorela M., Lavonius S., Salminen M., Vahlberg T., Viitanen M., Viikari L. (2020). Self-rated health and objective health status as predictors of all-cause mortality among older people: A prospective study with a 5-, 10-, and 27-year follow-up. BMC Geriatr..

[23] Fan Y., He D. (2022). Self-rated health, socioeconomic status and all-cause mortality in Chinese middle-aged and elderly adults. Sci. Rep..

[24] Torres-Collado L., de la Hera M.G., Compañ-Gabucio L.M., Oncina-Cánovas A., González-Palacios S., Notario-Barandiaran L., Vioque J. (2022). Self-reported health status and mortality from all-causes of death, cardiovascular disease and cancer in an older adult population in Spain. PLoS ONE.

[25] Zhang Y.L., Wu B.J., Chen P., Guo Y. (2021). The self-rated health status and key influencing factors in middle-aged and elderly: Evidence from the CHARLS. Medicine.

[26] Todorova I.L., Tucker K., Jimenez M.P., Lincoln A.K., Arevalo S.P., Falcón L.M. (2013). Determinants of self-rated health and the role of acculturation: Implications for health inequalities. Ethn. Health.

[27] Li Y., Nima Q., Yu B., Xiao X., Zeng P., Suolang D., He R., Ciren Z., Wangqing P., Laba C. (2021). Determinants of self-rated health among an older Tibetan population in a Chinese plateau area: Analysis based on the conceptual framework for determinants of health. BMC Public Health.

[28] de Oliveira T.L., de Oliveira R.V.C., Griep R.H., Moreno A.B., Almeida M.D.C.C.D., Almquist Y.B., Fonseca M.D.J.M.D. (2021). Brazilian Longitudinal Study of Adult Health (ELSA-Brasil) participant’s profile regarding self-rated health: A multiple correspondence analysis. BMC Public Health.

[29] Peduzzi P., Concato J., Kemper E., Holford T.R., Feinstein A.R. (1996). A simulation study of the number of events per variable in logistic regression analysis. J. Clin. Epidemiol..

[30] Concato J., Peduzzi P., Holford T.R., Feinstein A.R. (1995). Importance of events per independent variable in proportional hazards analysis I. Background, goals, and general strategy. J. Clin. Epidemiol..

[31] Campbell-Sills L., Stein M.B. (2007). Psychometric analysis and refinement of the connor–davidson resilience scale (CD-RISC): Validation of a 10-item measure of resilience. J. Trauma. Stress.

[32] Wang L., Shi Z., Zhang Y., Zhang Z. (2010). Psychometric properties of the 10-item Connor-Davidson Resilience Scale in Chinese earthquake victims. Psychiatry Clin. Neurosci..

[33] Cosco T.D., Kaushal A., Richards M., Kuh D., Stafford M. (2016). Resilience measurement in later life: A systematic review and psychometric analysis. Health Qual. Life Outcomes.

[34] Kwan Y.H., Ng A., Lim K.K., Fong W., Phang J.K., Chew E.H., Lui N.L., Tan C.S., Thumboo J., Østbye T. (2018). Validity and reliability of the ten-item Connor–Davidson Resilience Scale (CD-RISC10) instrument in patients with axial spondyloarthritis (axSpA) in Singapore. Rheumatol. Int..

[35] WHO World Health Organization. International Classification of Functioning, Disability and Health (ICF). GENEVA Web Site. Updated 2001. https://www.who.int/classifications/icf/en/.

[36] Ware J.E., Sherbourne C.D. (1992). The MOS 36-item short-form health survey (SF-36). I. Conceptual framework and item selection. Med. Care.

[37] Baron-Epel O., Kaplan G. (2001). General subjective health status or age-related subjective health status: Does it make a difference?. Soc. Sci. Med..

[38] Guilford J.P. (1956). Fundamental Statistics in Psychology and Education.

[39] Bellamy N., Buchanan W.W., Goldsmith C.H., Campbell J., Stitt L.W. (1988). Validation study of WOMAC: A health status instrument for measuring clinically important patient relevant outcomes to antirheumatic drug therapy in patients with osteoarthritis of the hip or knee. J. Rheumatol..

[40] Gandek B. (2015). Measurement Properties of the Western Ontario and McMaster Universities Osteoarthritis Index: A Systematic Review. Arthritis Care Res..

[41] Charlson M.E., Pompei P., Ales K.L., MacKenzie C.R. (1987). A new method of classifying prognostic comorbidity in longitudinal studies: Development and validation. J. Chronic Dis..

[42] Hoyl M.T., Alessi C.A., Harker J.O., Josephson K.R., Pietruszka F.M., Koelfgen M., Mervis J.R., Fitten L.J., Rubenstein L. (1999). Development and Testing of a Five-Item Version of the Geriatric Depression Scale. J. Am. Geriatr. Soc..

[43] Bumberger A., Borst K., Hobusch G.M., Willegger M., Stelzeneder D., Windhager R., Domayer S., Waldstein W. (2021). Higher patient knowledge and resilience improve the functional outcome of primary total knee arthroplasty. Wien. Klin. Wochenschr..

[44] Magaldi R.J., Staff I., Stovall A.E., Stohler S.A., Lewis C.G. (2019). Impact of Resilience on Outcomes of Total Knee Arthroplasty. J. Arthroplast..

[45] Nwankwo V.C., Jiranek W.A., Green C.L., Allen K.D., George S.Z., Bettger J.P. (2021). Resilience and pain catastrophizing among patients with total knee arthroplasty: A cohort study to examine psychological constructs as predictors of post-operative outcomes. Health Qual. Life Outcomes.

[46] Majnarić L.T., Bosnić Z., Guljaš S., Vučić D., Kurevija T., Volarić M., Martinović I., Wittlinger T. (2021). Low Psychological Resilience in Older Individuals: An Association with Increased Inflammation, Oxidative Stress and the Presence of Chronic Medical Conditions. Int. J. Mol. Sci..

[47] Burton N.W., Pakenham K.I., Brown W.J. (2009). Evaluating the effectiveness of psychosocial resilience training for heart health, and the added value of promoting physical activity: A cluster randomized trial of the READY program. BMC Public Health.

[48] Szanton S.L., Gill J.M. (2010). Facilitating resilience using a society-to-cells framework: A theory of nursing essentials applied to research and practice. ANS Adv. Nurs. Sci..

[49] Tavakol M., Dennick R. (2011). Making sense of Cronbach’s alpha. Int. J. Med. Educ..

